# Hemostasis via an endoscopic full-thickness suturing device with extended cap length method and red dichromatic imaging for deep colonic diverticular bleeding

**DOI:** 10.1055/a-2767-0168

**Published:** 2026-01-13

**Authors:** Takahiro Muramatsu, Masakatsu Fukuzawa, Fumito Yamanishi, Makoto Arashiyama, Fumi Naruse, Tomohiro Kaketani, Takao Itoi

**Affiliations:** 1 38548 Department of Gastroenterology and Hepatology, Tokyo Medical University Hospital, Tokyo, Japan

**Fig. 1:**
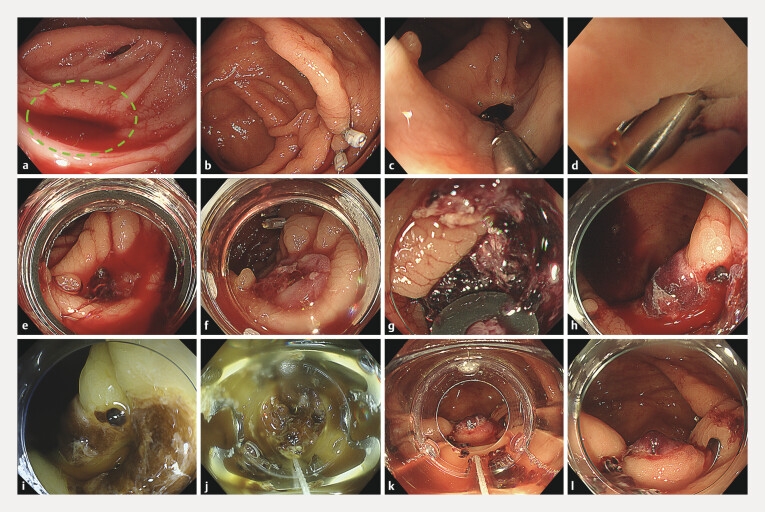
Endoscopic images. **a** Active bleeding was observed from a diverticulum in the ascending colon (green dotted circle). **b** A marking clip was placed near the responsible diverticulum. **c** A clip was inserted into the diverticulum, which yielded hemostasis. **d** Primary hemostasis was achieved; however, the diverticulum was so deep that the clips were hidden. **e** Rebleeding was observed from the previously clipped diverticulum. **f** Endoscopic band ligation yielded temporary hemostasis. **g** During the third colonoscopy for recurrent bleeding, we discovered that the previously applied band had detached. **h** An endoscopic image under white-light imaging. **i** An endoscopic image under red dichromatic imaging. **j** The entire diverticulum was suctioned into an OTS-clip via an extended cap length method. **k** The OTS-clip was successfully deployed. **l** The OTS-clip was firmly fixed, resulting in successful hemostasis.

**Fig. 2:**
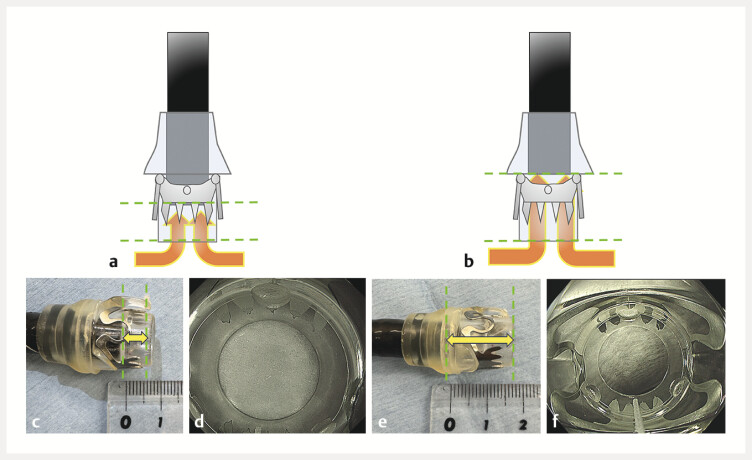
Schema of an OTS-clip deployed using an extended cap length method. **a** Schema of the normal deployment of the OTS-clip. **b** Appearance of the normal deployment of the OTS-clip. The cap length (from the endoscope tip to the tip of the OTS-clip cap) is 6 mm. **c** An endoscopic view of the normal deployment of the OTS-clip. **d** Schema of the OTS-clip deployed via the extended cap length method (ECLM). **e** Appearance of the OTS-clip deployed via the ECLM. The cap length (from the endoscope tip to the tip of the OTS-clip cap) is 12 mm. **f** An endoscopic view of the OTS-clip deployed via the ECLM. The endoscopic view is slightly narrower than that with normal deployment, but it does not interfere with the procedure.

Diverticular bleeding accounts for approximately 60% of cases of acute lower gastrointestinal bleeding and is a common disease 1 . Endoscopic hemostasis is mainly achieved using endoclips or endoscopic band ligation (EBL), although rebleeding may occur. For such cases, an OTS-clip has been reported as an effective option 2 3 . Furthermore, red dichromatic imaging (RDI) can facilitate the identification of the diverticular bleeding site 4 5 . Herein, we describe a case of recurrent diverticular bleeding successfully treated by modifying the OTS-clip setup with an extended cap length method (ECLM) to increase the suction depth and by using RDI to improve the visualization of the bleeding site ( Video 1 ). A 74-year-old man who was taking aspirin for essential thrombocythemia was presented with hematochezia. Computed tomography revealed multiple diverticula, and upon emergency colonoscopy, we discovered active bleeding from a diverticulum of the ascending colon. After marking clips were fixed near the diverticulum, hemostasis was achieved via clipping; however, the diverticulum was so deep that the clips were hidden ( Fig. 1 **a–d** ). Rebleeding occurred the following day, and EBL yielded temporary hemostasis ( Fig. 1 **e, f** ), but bleeding recurred several hours later. As both clipping and EBL had failed, hemostasis via an OTS-clip was planned during third colonoscopy. Because the diverticulum was deep and the band had detached ( Fig. 1 **g** ), the OTS-clip was attached with an extended cap to increase the suction width ( Fig. 2 ). RDI was used to enhance the visibility of the bleeding site within the diverticulum. The diverticulum was fully inverted into the elongated OTS-clip cap, and the clip was deployed, resulting in complete hemostasis ( Fig. 1 **h–l** ). No further bleeding occurred.

Hemostasis achieved via an OTS-clip with an extended cap length method and red dichromatic imaging for bleeding from a deep colonic diverticulum.Video 1

In conclusion, an OTS-clip attached via the ECLM enabled the complete inversion and reliable hemostasis of a deep diverticulum. Combined with RDI, this approach may be useful as treatment for refractory diverticular bleeding.

Endoscopy_UCTN_Code_TTT_1AQ_2AZ
